# Long-term effects of early sports participation on health-related quality of life: a cross-sectional study in Finland

**DOI:** 10.1093/eurpub/ckag018

**Published:** 2026-02-06

**Authors:** Tuomas S M Lehtola, Päivi Korhonen, Niko Wasenius, Hannu Kautiainen, Merja K Laine

**Affiliations:** Department of General Practice and Primary Health Care, University of Helsinki and Helsinki University Hospital, Helsinki, Finland; Department of General Practice, University of Turku, Turku, Finland; Wellbeing Services County of Southwest Finland, Turku, Finland; Department of General Practice and Primary Health Care, University of Helsinki and Helsinki University Hospital, Helsinki, Finland; Folkhälsan Research Centre, Helsinki, Finland; Department of General Practice and Primary Health Care, University of Helsinki and Helsinki University Hospital, Helsinki, Finland; Folkhälsan Research Centre, Helsinki, Finland; Primary Health Care Unit, Kuopio University Hospital, Kuopio, Finland; Department of General Practice and Primary Health Care, University of Helsinki and Helsinki University Hospital, Helsinki, Finland; Folkhälsan Research Centre, Helsinki, Finland

## Abstract

The effects of sport participation (SP) are typically assessed over relatively short time frames, with limited information regarding long-term impacts. The aims of this study were to evaluate the relationship of SP during childhood or adolescence with physical activity (PA) and health-related quality of life (HRQoL) in adulthood. We performed an observational cross-sectional study. A population survey was conducted in two rural Finnish towns during 2005–7. Apparently healthy cardiovascular risk subjects aged 45–70 years were identified, and information regarding current PA and HRQoL (36-item Short Form Health Survey) and childhood or adolescent SP were gathered using questionnaires. Current PA was measured in metabolic equivalent hours per week (MET-h/week). Participants (*n* = 2503; mean age 58 years) were divided into three groups based on their SP levels during childhood or adolescence: none (*n* = 338), hobby (*n* = 1713), and competitive (*n* = 452). The mean level of current PA was 8.1 (SD 6.8) MET-h/week in none group, 9.2 (7.1) in hobby group, and 10.4 (7.8) in competitive group (*P < .*001). Participants in the competitive group reported significantly better HRQoL compared to other groups (*P < .*001). Childhood or adolescent SP modified the relationship between adulthood PA and HRQoL. Individuals with high levels of SP during childhood or adolescence were more likely to remain active in adulthood. SP in early life modified the association between adulthood PA and HRQoL and was also associated with better HRQoL in adulthood especially in mental health-related domains. These findings highlight the importance of promoting PA from an early age.

## Introduction

In the modern world, various technological advancements and changes in society have caused declines in the amount of physical activity (PA) experienced during work and transportation [1]. With increasing occupational and leisure time spent online and being inactive, actions to promote PA and reduce sedentary lifestyles are needed. Reliable information regarding the health benefits of PA is required for interventions to be effective.

PA is known to provide various health benefits. Regular PA reduces the risk of more than 25 chronic medical conditions, including cardiovascular diseases (CVD), diabetes, osteoporosis, and cancer [2, 3]. Through a range of known and partially unknown biological mechanisms, PA has the potential to extend the human lifespan by years, and it is associated with lower all-cause mortality [3–5]. While PA is a broad concept that can be interpreted to refer to nearly any kind of movement, sport participation (SP) is a more distinct term involving organized sports and is thus easier to measure via questionnaires. Both terms have been used in the research literature.

SP during childhood and adolescence appears to predict higher PA in adulthood. A recent systematic review identified the frequency and persistence of SP to associate with higher PA levels in adulthood and concluded that a moderate to strong relationship exists between SP during childhood and adolescence and PA in adulthood [6]. Many possible measures exist for assessing the health effects of PA, with health-related quality of life (HRQoL) being one example that focuses on subjective health perceptions.

HRQoL is a multidimensional and subjective concept encompassing physical, psychological, and social components related to diseases and treatments [7]. HRQoL reflects an individual’s overall health status: good HRQoL predicts better overall health, while poor HRQoL is associated with an increased risk of hospitalization and mortality [8, 9]. PA has been found to predict HRQoL across various age groups [10–12]. Previous study findings suggest that SP during childhood and adolescence is associated with better HRQoL, at least in young men (mean age of 26 years) and particularly in the mental domain [13]. However, research on the long-term associations of childhood or adolescent SP with HRQoL and PA in adulthood is lacking [13].

Our aim was to evaluate the relationship between SP during childhood or adolescence and HRQoL in adulthood. We had the opportunity to investigate this in a CVD risk population. Additionally, we investigated how childhood or adolescent SP influences PA in adulthood and whether the association between adulthood PA and HRQoL is modified by SP levels during childhood or adolescence. We hypothesized that childhood or adolescent SP would have a positive impact on the abovementioned factors.

## Methods

### Study subjects and design

We performed an observational cross-sectional study. Study participants were selected from a CVD risk assessment programme (the HARMONICA project—Harjavalta Risk Monitoring for Cardiovascular Disease) conducted in the small Finnish towns of Harjavalta and Kokemäki between 2005 and 2007. All 6013 inhabitants aged 45–70 years were mailed a CVD risk assessment questionnaire, a type 2 diabetes risk assessment form (Finnish Diabetes Risk Score, FINDRISC) [14], and a measuring tape for waist circumference. Those intending to participate were asked to return the completed questionnaires by post, which was done by 74% (*n* = 4450) of the initially contacted persons. Individuals with previously diagnosed diabetes, CVD, or chronic kidney disease were omitted from this cohort.

The mailed questionnaires assessed risk factors for CVD. Subjects were asked to report their waist circumference measured at the navel level during exhalation, their use of antihypertensive drugs, their most recent blood pressure measurement, their history of gestational diabetes and/or gestational hypertension, along with any parental or sibling history of myocardial infarction and stroke. Subjects with one or more of these risk factors or a high FINDRISC score (≥12 in Harjavalta, ≥15 in Kokemäki) were invited to attend further examinations. These included a general health survey, laboratory tests, and a physical examination performed by a trained nurse.

Of the invited participants, 2503 completed the questionnaire regarding childhood or adolescent SP and current PA, and they thus comprise the subjects of this study. All the study subjects were of ethnic Finnish origin. Childhood or adolescent SP was assessed with the question “Have you engaged in sports during your childhood or youth?” with three response options: “no,” “yes, as a hobby,” “yes, competitive sports.” The subjects were divided into three groups based on their responses: none (*n* = 338), hobby (*n* = 1713), and competitive (*n* = 452).

### Questionnaires

#### Health-related quality of life

The Short Form Health Survey (SF-36) was used to assess HRQoL[15]. SF-36 measures eight domains of HRQoL: (i) physical functioning, (ii) role limitations due to physical health, (iii) bodily pain, (iv) general health, (v) mental health, (vi) role limitations due to emotional problems, (vii) social functioning, and (viii) vitality. For each domain, a score ranging from 0 to 100 was calculated, where 0 represents the poorest and 100 the best possible health status. Self-rated health (SRH) was assessed using the first question in the SF-36: “How would you rate your health?” with five response options ranging from excellent (1) to poor (5).

#### Adulthood physical activity

Metabolic equivalent hours per week (MET-h/week) values were estimated from the questionnaire responses. Participants were asked the following three questions to capture their current PA levels: (i) “How many minutes per day do you walk or bike during your work commute?” (ii) “How often do you exercise for at least 30 minutes during your leisure-time?” and (iii) “If you exercise, how strenuous is it generally?” To calculate the weekly levels of commuting PA in MET-hours, we multiplied the estimated number of days that individuals worked per week (=5 days per week) with the estimated average intensity of commuting activities (3 METs) and with duration (0 min =“not working or using a motor vehicle,” 7.5 min =“less than 15 minutes,” 22 min = “15–29 minutes,” 44.5 min = “30–59 minutes,” 60 min = “60 minutes or over”). For leisure time physical activity (LTPA), we calculated the average level of LTPA by multiplying duration (30 min), frequency (0 = never, 0.25 = less than once a month, 0.375 = 1–2× per month, 1 = once a week, 2.5 = 2–3× a week, 5 = 4–6× a week, or 7 = everyday) and intensity in METs (3 = no sweating/not out of breath, 4.5 = out of breath and some sweating, and 6 = out of breath and much sweating). After these calculations, we summed the levels of commuting PA and LTPA to calculate current adulthood PA. For the analyses, we subdivided each childhood or adolescent SP group into two categories based on adulthood PA measured in MET-h/week relative to the study population median (7.5 MET-h/week): MET ≥ median and MET < median, resulting in six groups.

#### Depressive symptoms

Depressive symptoms were assessed using the 21-item Beck Depression Inventory 2 (BDI) survey [16]. BDI consists of 21 questions, each scored on a scale from 0 to 4.

#### Lifestyle factors

Alcohol consumption was assessed using the Alcohol Use Disorder Identification Test (AUDIT) [17]. Subjects were also asked to report their smoking history and were divided into current smokers and non-smokers based on their responses.

#### Sociodemographic factors

Information regarding subjects’ education duration, cohabiting, and occupational status (blue-collar, white-collar, retired) was collected through the self-administered questionnaires.

### Clinical measurements

#### Oral glucose tolerance test

Subjects were instructed to fast for at least 12 hours before the laboratory tests. Fasting plasma glucose and 2-hour plasma glucose samples were measured from capillary blood using the HemoCue Glucose 201+ system after ingestion of 75 g of anhydrous glucose dissolved in water.

#### Lipids

Total cholesterol, high-density lipoprotein cholesterol (HDL-C), and triglycerides were measured enzymatically using the Olympus AU64 system (Japan), while low-density lipoprotein cholesterol (LDL-C) was calculated using Friedewald’s formula. Non-HDL cholesterol was calculated by subtracting HDL-C from the total cholesterol.

#### Physical measurements

Blood pressure (mmHg) was measured using a mercury sphygmomanometer after subjects rested in a sitting position for ≥5 minutes, with the cuff placed around the upper arm. Blood pressure was measured twice, and the mean of the two values was recorded. Systolic and diastolic blood pressure were defined according to Korotkoff sounds I and V, respectively. Height and weight of the subjects were measured in a standing position. Height was recorded to the nearest 0.5 cm and weight to the nearest 0.1 kg using a digital scale (Seca 861). Body mass index (BMI) was calculated as weight (kg) divided by the square of the height (m^2^).

#### Drug use

Drug use was assessed from the questionnaire and medical records.

#### Statistics

Descriptive statistics were presented as means with standard deviations (SD) or as values with percentages. The relationship between childhood or adolescent SP levels and adult PA levels was statistically assessed using two-way analysis of variance models or logistic models. The models included the main effects (SP and PA) and the interaction effects between them. The analyses were adjusted for sex, age, smoking, alcohol use, BMI, and length of education (in years) when appropriate. In cases where the assumptions were violated (non-normality), a bootstrap- or permutation-type test was used. Normal distributions were evaluated graphically using the Shapiro–Wilk W test. Effect size (*d*) was calculated using the method by Cohen, adjusted for sex, age, smoking, alcohol use, BMI, and length of education (in years). A Cohen effect size of 0.20 is considered small, 0.50 is moderate, and 0.80 is large. Hommel’s procedure was applied to correct levels of significance for multiple testing. All statistical analyses were performed using STATA 18.0 (STATA Corp., College Station, TX, USA).

#### Ethical aspects

The study protocol and consent forms were reviewed and approved by the Ethics Committee of the Satakunta Hospital District, Finland. All participants provided written informed consent for the project and subsequent research.

## Results

### Characteristics of study population

**Figure 1.**
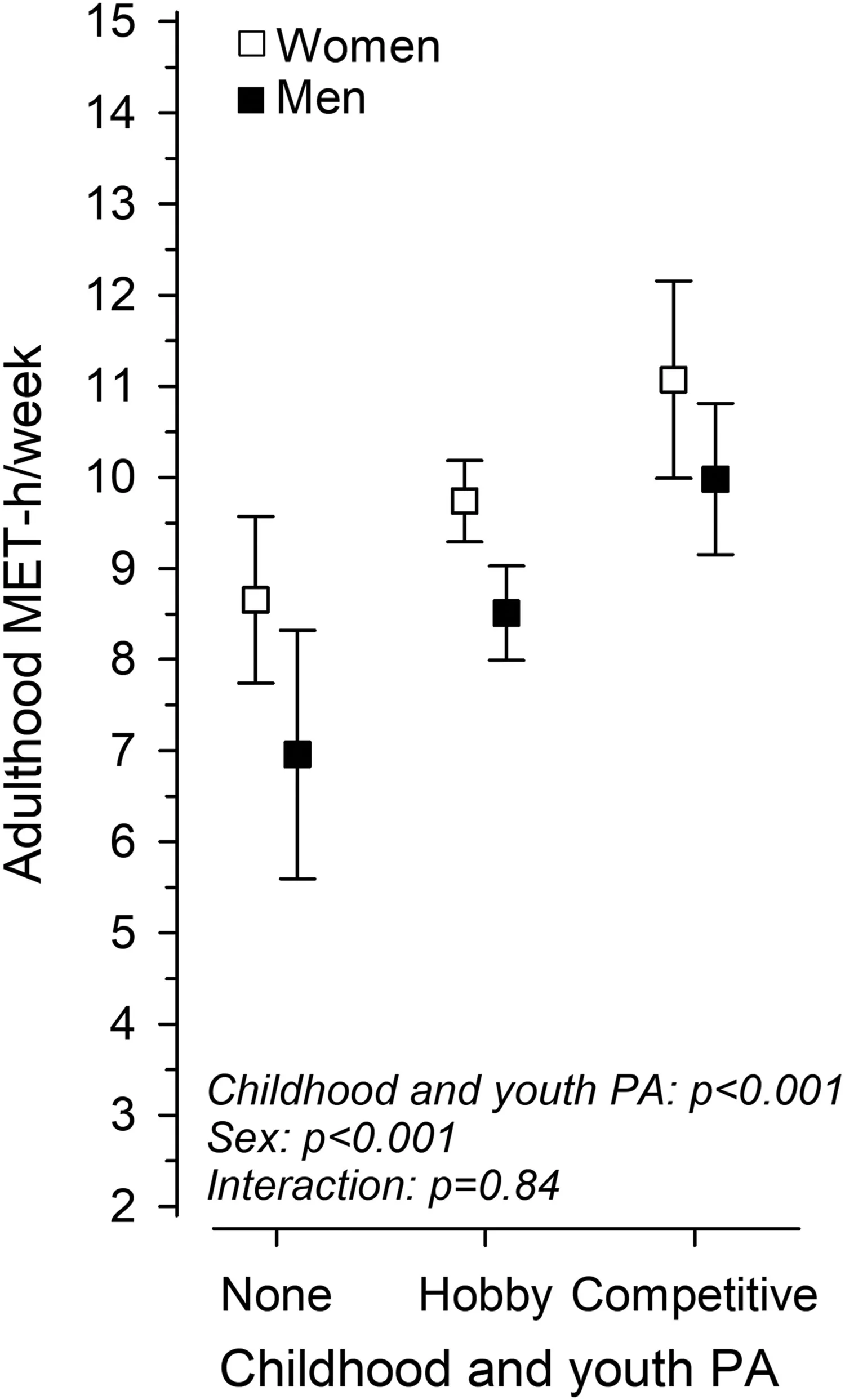
Association of mean adulthood physical activity (PA) level and childhood or adolescent Sport participation (SP) in men and women.

Table 1 presents the baseline characteristics of the study subjects (*n* = 2503), categorized into six groups. Subjects with high SP levels during childhood or adolescence were more likely to be male and employed in blue-collar occupations. Childhood or adolescent SP was further associated with a higher prevalence of current smoking and higher diastolic blood pressure. Moreover, childhood or adolescent SP was associated with decreased depressive symptoms and lower HDL-C levels in adulthood. Adulthood PA was further associated with a lower prevalence of smoking and lower diastolic blood pressure. Moreover, adulthood PA was associated with higher HDL-C levels and decreased depressive symptoms. Figure 1 displays adulthood PA levels according to SP levels during childhood or adolescence. A statistically significant positive association was observed between childhood or adolescent SP and adulthood PA. Women had significantly higher levels of PA than men did in adulthood.

**Table 1.** Characteristics of the study population according to childhood or adolescent sports participation (SP) levels (none, hobby, competitive) and adulthood physical activity (PA) levels (≤median and >median).

Childhood/adolescent SP	None		Hobby		Competitive		*P* value		
Adulthood PA	MET-h/week ≤7 (*N* = 186)	MET-h/week ≥8 (*N* = 152)	MET-h/week ≤7 (*N* = 846)	MET-h/week ≥8 (*N* = 867)	MET-h/week ≤7 (*N* = 203)	MET-h/week ≥ 8 (*N* = 249)	Childhood	MET-h/week	Interaction
Women, *n* (%)	117 (63)	116 (76)	454 (54)	533 (61)	68 (33)	99 (40)	<.001	<.001	.43
Age, mean (SD)	57 (7)	58 (7)	58 (7)	58 (7)	57 (7)	58 (7)	.45	.002	.57
Length of education (in years), mean (SD)	10.7 (2.9)	10.5 (3.1)	10.5 (2.7)	10.2 (2.7)	10.5 (2.7)	10.6 (2.5)	.18	.41	.55
Relationship, *n* (%)	139 (75)	111 (73)	675 (80)	671 (77)	163 (80)	199 (80)	.092	.48	.90
Occupational status							.004	.021	.096
Blue-collar	95 (51)	69 (45)	465 (55)	413 (48)	108 (53)	136 (55)			
White-collar	24 (13)	13 (9)	39 (5)	56 (6)	15 (7)	11 (4)			
Retired	67 (36)	70 (46)	342 (40)	398 (46)	80 (39)	102 (41)			
SRH, *n* (%)							.27	<.001	.009
Poor	87 (47)	60 (39)	359 (42)	306 (35)	99 (49)	71 (29)			
Good	62 (33)	41 (27)	247 (29)	283 (33)	58 (29)	78 (31)			
Excellent	37 (20)	51 (34)	240 (28)	278 (32)	46 (23)	100 (40)			
Smokers, *n* (%)	28 (15)	19 (13)	161 (19)	121 (14)	59 (29)	57 (23)	<.001	.026	.90
AUDIT, mean (SD)	3.9 (4.4)	3.8 (4.2)	5.0 (5.3)	4.1 (4.3)	6.1 (5.8)	5.7 (5.1)	<.001	.053	.31
BMI, kg/m^2^ mean (SD)	29.8 (5.5)	28.2 (5.3)	29.5 (5.0)	28.0 (4.6)	29.4 (5.6)	28.4 (4.2)	.59	<.001	.59
BDI, mean (SD)	6.7 (6.2)	6.2 (5.9)	6.3 (5.8)	5.9 (5.3)	6.0 (5.5)	4.7 (4.4)	.013	.009	.32
BP, mmHg, mean (SD)									
Systolic	138 (17)	139 (19)	140 (19)	140 (19)	143 (18)	141 (19)	.042	.53	.43
Diastolic	84 (10)	82 (10)	85 (10)	84 (10)	87 (11)	85 (9)	<.001	<.001	.21
Glucose, mmol/l, mean (SD)									
Fasting	5.63 (1.46)	5.47 (0.88)	5.66 (1.25)	5.54 (0.93)	5.77 (1.28)	5.61 (1.16)	.20	.011	.89
2 h	7.51 (2.26)	7.17 (1.90)	7.58 (2.40)	7.29 (2.09)	7.58 (2.52)	6.88 (1.95)	.23	<.001	.53
Cholesterol, mean, mmol/l (SD)	5.37 (1.00)	5.37 (1.00)	5.37 (1.00)	5.41 (0.95)	5.36 (0.93)	5.38 (1.01)	.91	.65	.93
LDL, mmol/l, mean (SD)	3.28 (0.90)	3.13 (0.87)	3.24 (0.90)	3.25 (0.85)	3.26 (0.88)	3.25 (0.91)	.73	.27	.33
Non-HDL, mmol/l, mean (SD)	3.85 (1.01)	3.70 (0.96)	3.87 (1.00)	3.82 (0.92)	3.89 (0.93)	3.82 (1.01)	.47	.075	.68
HDL, mmol/l, mean (SD)	1.52 (0.45)	1.67 (0.44)	1.50 (0.44)	1.59 (0.43)	1.47 (0.40)	1.56 (0.51)	.032	<.001	.46
Triglycerides, mmol/l, mean (SD)	1.40 (0.72)	1.29 (0.74)	1.48 (0.82)	1.31(0.64)	1.50 (0.85)	1.35 (0.69)	.29	<.001	.76
Medication, *n* (%)									
Lipids	19 (10)	20 (13)	109 (13)	118 (14)	24 (12)	30 (12)	.60	.43	.81
Blood pressure	62 (33)	44 (29)	303 (36)	282 (33)	70 (34)	80 (32)	.55	.16	.95
Antidepressants	14 (8)	7 (5)	38 (4)	25 (3)	10 (5)	7 (3)	.16	.035	.97

Two-way ANOVA and logistic models were used for statistical testing.

AUDIT, Alcohol Use Disorder Identification Test; BMI, body mass index; BDI, Beck’s Depression Inventory; BP, blood pressure; HDL-C, high density lipoprotein; LDL-C, low density lipoprotein; MET-h/wk, metabolic equivalent hours per week; PA, physical activity; SP, sports participation; SRH, self-rated health.

### Health-related quality of life

**Figure 2.**
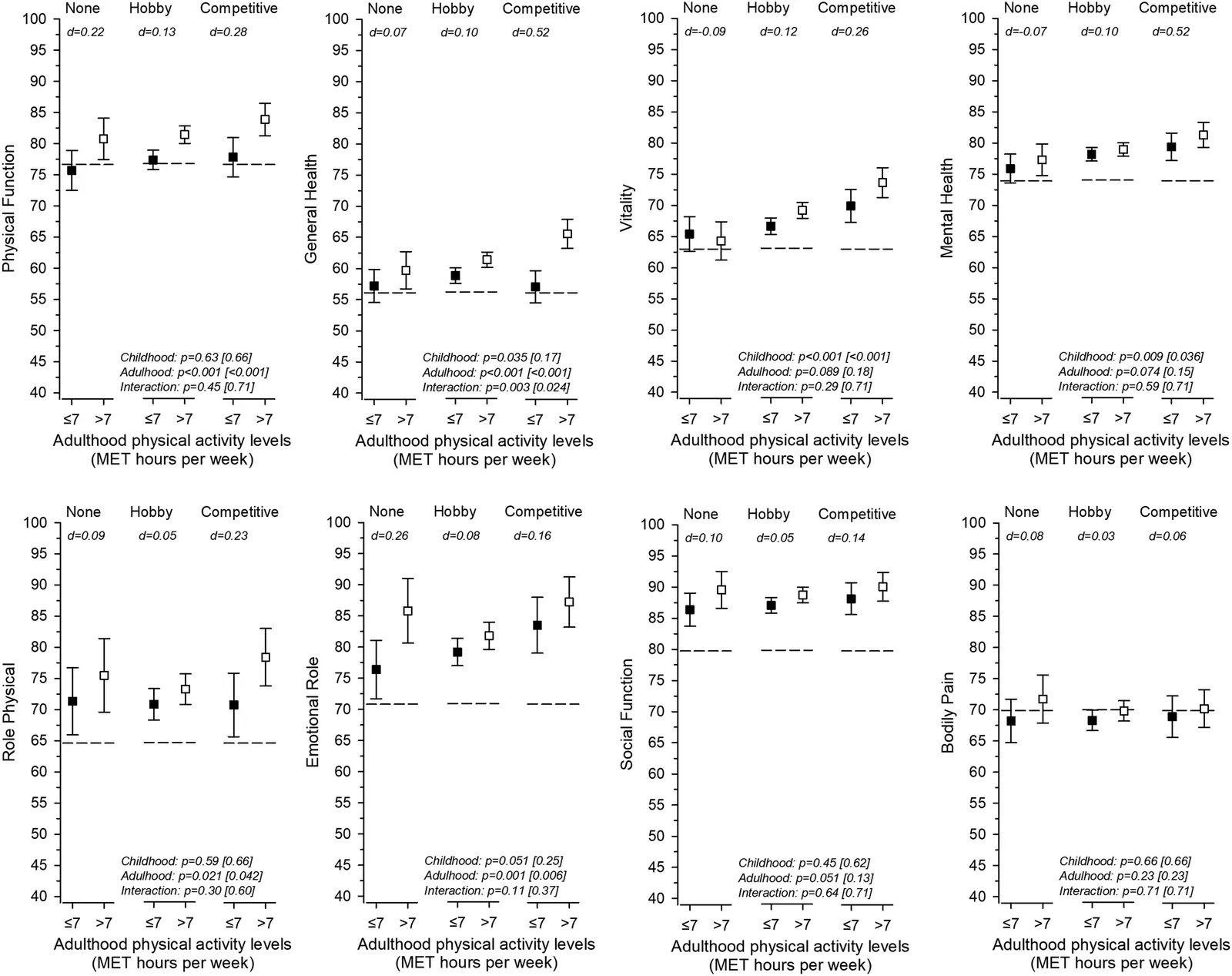
Health-related quality of life dimensions (Short Form Health Survey SF-36) according to childhood or adolescent sports participation levels (none, hobby, competitive) and adulthood physical activity levels. *P* values assessed using two-way analysis of variance models and adjusted using for sex, age, smoking, use of alcohol, body mass index, and length of education (in years). Adjusted Cohen’s effect size (d); Hommel’s corrected probabilities (multiplicity). Abbreviation: MET, metabolic equivalent.

Figure 2 presents the adjusted SF-36 scores. A significant statistical interaction between childhood or adulthood PAs was present only for general health after adjusting for sex, age, smoking, and alcohol use, BMI, and length of education (in years). Both PA in adulthood and SP during childhood or adolescence were positively associated with general health. Childhood or adolescent SP was also positively associated with mental health and vitality. Adulthood PA additionally showed a positive association with physical function, physical role, and emotional role.

## Discussion

Our results showed that SP during childhood or adolescence was positively associated with PA in adulthood. While adulthood PA was associated with high HRQoL in most physical domains, childhood or adolescent SP was associated with higher adulthood HRQoL in mental health-related domains (the mental health and vitality domains). Furthermore, childhood or adolescent SP was found to modify the relationship between adulthood PA and HRQoL: the increase in the general health domain score between adulthood inactive and active subgroups was highest for subjects exhibiting the most childhood or adolescence SP.

Previous literature has reported a positive relationship between PA and HRQoL in children and adolescents as well as adults [10, 11]. In a recent systematic review, PA was found to associate with better HRQoL in the physical, mental, and social domains in the general population of children and adolescents [10]. Another systematic review found a positive association between PA and various HRQoL domains in the general adult population. This effect was reported to be strongest in the physical functioning and vitality domains of HRQoL [11]. The present study found high levels of childhood or adolescent SP to associate with significantly better HRQoL in adulthood. This association was strongest in the mental HRQoL domains. No significant associations in the physical domains were seen, except for general health. The positive association between early-age SP and adulthood HRQoL could be explained by adulthood PA to some extent mediating this effect or by early-age SP having an effect independent of adulthood PA status. Since the association was also seen in subjects with low levels of adulthood PA, childhood or adolescent SP may potentially have independent, long-term effects. Direct conclusions regarding the mechanisms behind the observed relationship between early-age SP and adulthood mental HRQoL cannot be drawn from our study results due to the observational cross-sectional study design. However, the potential beneficial effect of childhood or adolescent SP on mental HRQoL may possibly be partly mediated by learning social skills and interacting with others in organized sports [13]. In a previous study that analyzed young athletes, team sports athletes reported less depression and anxiety than did individual sports athletes [18]. Since sports participation is valued by society, it may improve self-esteem which may also contribute to better HRQoL. Multiple studies have reported better self-perception in competitive athletes compared with noncompetitive subjects [19, 20]. Various studies have indicated adolescence to be a critical period for social development [21, 22]. Thus, the social interaction and experiences formed during that time may play a part in how individuals function in adulthood, ultimately reflecting mental HRQoL.

While the present study found high levels of childhood or adolescent SP to associate with higher scores in the vitality and mental health domains of adulthood HRQoL, differing results were reported in a study that found no association between adolescent PA and adulthood HRQoL [23]. This lack of observed relationship could be explained by the small sample size (*n* = 310) and the younger mean age of participants at follow-up (men 36 years; females 35 years) [23]. However, a more recent Finnish study reported results consistent with ours. They found SP at the age of 12 years to associate with better HRQoL in young men (mean age of 26 years), particularly in the mental health domains [13]. As noted by Appelqvist-Schmidlechner *et al.* [13], PA in childhood may contribute more to mental HRQoL in adulthood while physical HRQoL depends more on current PA status. Consistently with previous studies, our present study also found current PA status (PA in adulthood) to positively associate with HRQoL in various domains [10, 11]. Subjects were considered physically active if they had adulthood PA levels above the study population median (7.5 MET-h/week), which corresponds to the lower end of the common recommendations of 150–300 min of moderate-intensity PA/week, when moderate-intensity PA is defined as ≥3 METs [24]. In their systematic review, Bize *et al.* [11] note that some conceptual overlap may exist between self-reported PA levels and physical HRQoL, which may partly explain the strong association between current PA status and physical HRQoL levels. Finally, in the present study, we found childhood or adolescent SP to modify the relationship between adulthood PA and HRQoL in the general health domain. Subjects with the highest levels of early-age SP (competitive group) showed the highest increase in the general health score between the adulthood inactive and active subgroups compared with subjects in the hobby and none groups. In our study, the differences in SF-36 domain scores between consecutive childhood or youth SP groups was relatively small: the difference in HRQoL domain scores between the highest and lowest early-age SP groups was 10 points at most. On a population level, even small changes are meaningful. On an individual level, a difference of 3–5 points in the SF-36 scores is considered clinically important [25].

Our study subjects with high levels of childhood or adolescent SP exhibited higher levels of PA in adulthood, which is consistent with previous studies [6, 26]. Other long-term effects of early-age SP have also been investigated. Previous studies have indicated that childhood PA may be inversely related to adulthood depression [27]. This is also consistent with our results since we found high levels of childhood or adolescent SP to associate with low BDI scores in adulthood. The positive long-term health benefits of early-age SP may contribute to better HRQoL, as also observed in this study.

### Strengths and limitations

To our knowledge, this is the first study to simultaneously assess the relationship between childhood or adolescent SP, adulthood PA, and HRQoL. We used SF-36 to measure HRQoL, which is one of the most used items for assessing HRQoL and increases the generalizability of our results. Furthermore, our study sample was relatively large and had a long follow-up time. Certain limitations also need to be addressed. MET-hours were calculated based on the available questionnaire responses and represent only rough estimates. Childhood or adolescent SP was assessed with a single question, making it a crude estimate of the variable. Accuracy could have been improved by additional questions assessing SP frequency, intensity, and type. Our study was observational in nature, meaning causality cannot be deduced. Reverse causality could potentially explain the results, at least partly, since childhood and adolescent health impairment, both physical and mental, have been associated with lower PA levels [28, 29]. Voluntary participation via mail may result in selection bias, for example if only people with a certain HRQoL level participate. Studies have demonstrated that people who choose to participate in studies tend to have higher socioeconomical status and are more likely to be employed and female [30]. These factors may have led to falsely high HRQoL levels in the study population. PA, SP, and many other baseline characteristics were self-reported, which makes results susceptible to social desirability and recall biases, especially in the case of childhood SP. These biases may have led to falsely high levels of reported PA and SP levels, since health-promoting PA is socially valued in Finland [31]. Furthermore, objective measurements of PA, such as accelerometers, have been associated with better HRQoL than have subjective measures [32], which may indicate that people tend to overestimate their PA levels. Finally, we note that this study was conducted in Finland, where children and adolescents have equivalent opportunities to participate in sport activities despite socioeconomical differences, which should be kept in mind when interpreting the study results.

## Conclusions

In the present study, we found that high levels of SP during childhood or adolescence were positively associated with both PA and HRQoL (particularly the mental health domains) in adulthood. Childhood or adolescent SP also modified the relationship between adulthood PA and HRQoL: the increase in the general health domain score between adulthood inactive and active subgroups was highest for subjects with the most childhood or adolescent SP. These findings suggest that initiating PA at an early age may yield the greatest long-term subjective health perceptions. However, since the study design was observational and cross-sectional, direct long-term associations cannot be drawn. Children and adolescents represent an effective target group for PA-promoting interventions, as PA levels tend to track into adulthood to some extent. Measures to promote PA in children through schools and extracurricular activities such as sport clubs are likely to have lasting health effects and should be implemented.

Conflict of interest: None declared.

## Data Availability

The data underlying this article will be shared on reasonable request. Key points Sports participation (SP) during childhood or adolescence was associated with better health-related quality of life (HRQoL) in adulthood, particularly in the mental health domains of SF-36 SP during childhood or adolescence modified the relationship between physical activity (PA) and HRQoL in adulthood: early-age SP was associated with greater HRQoL benefits from PA in adulthood Subjects with higher SP levels during childhood or adolescence were also more likely to have higher PA levels in adulthood Promoting PA in early life may provide long-lasting health benefits: therefore, effective measures should be implemented to support PA in children and adolescents
